# Strong Wind Characteristics and Buffeting Response of a Cable-Stayed Bridge under Construction

**DOI:** 10.3390/s20041228

**Published:** 2020-02-24

**Authors:** Lei Yan, Lei Ren, Xuhui He, Siying Lu, Hui Guo, Teng Wu

**Affiliations:** 1 School of Civil Engineering, Central South University, Changsha 410075, China; leiyan@csu.edu.cn (L.Y.); leiren@csu.edu.cn (L.R.); csulusiying@163.com (S.L.); 2 Railway Engineering Research Institute, China Academy of Railway Sciences, Beijing 100081, China; superhugo@163.com; 3 Department of Civil, Structural and Environmental Engineering, University at Buffalo, State University of New York, Buffalo, NY 14126, USA; tengwu@buffalo.edu

**Keywords:** wind and structural health monitoring, wireless sensor networks, field measurement, cable-stayed bridge, wind characteristics, buffeting response, construction

## Abstract

This study carries out a detailed full-scale investigation on the strong wind characteristics at a cable-stayed bridge site and associated buffeting response of the bridge structure during construction, using a field monitoring system. It is found that the wind turbulence parameters during the typhoon and monsoon conditions share a considerable amount of similarity, and they can be described as the input turbulence parameters for the current wind-induced vibration theory. While the longitudinal turbulence integral scales are consistent with those in regional structural codes, the turbulence intensities and gust factors are less than the recommended values. The wind spectra obtained via the field measurements can be well approximated by the von Karman spectra. For the buffeting response of the bridge under strong winds, its vertical acceleration responses at the extreme single-cantilever state are significantly larger than those in the horizontal direction and the increasing tendencies with mean wind velocities are also different from each other. The identified frequencies of the bridge are utilized to validate its finite element model (FEM), and these field-measurement acceleration results are compared with those from the FEM-based numerical buffeting analysis with measured turbulence parameters.

## 1. Introduction

Strong wind gusts may cause a severe dynamic response of long-span bridges. The field measurement of wind characteristics plays an important role in accurately modeling wind effects on bridges. Thus, the field measurement based on a wind monitoring system (WMS) is of great significance for engineers to take full advantage of the realistic wind environment for bridges. To obtain the full-scale databases of wind characteristics for a long-span bridge, a WMS is usually installed at the measurement tower near the bridge [1,2,3,4]. However, the wind turbulence parameters may exhibit vast variability between field measurements at the measurement tower on lands and the bridge over lakes and seas [5,6]. Compared to the land-based measurement tower, the WMS installed on the bridge is apparently more representative and accurate. Although the WMS has become very popular and been treated as an essential part of the united wind and structural health monitoring system (WSHMS) in major and important bridges around the world to enhance structural safety and verify the current wind-induced vibration theory [7,8,9,10,11,12,13,14,15,16,17,18], most of the available studies concentrate on the wind characteristics and buffeting response of cable-supported bridges under the service stage. On the other hand, it is well known that the cable-stayed bridges are considerably more vulnerable to oncoming wind turbulence during construction than after completion [8,18,19,20,21,22,23].

Although comprehensive numerical analyses have appeared for wind-induced vibrations of long-span bridges under construction, they require variously identified bridge aerodynamic force parameters of deck or tower, such as eighteen flutter derivatives and six-component aerodynamic admittances of bridge decks from the wind-tunnel testing of a sectional model or the computational fluid dynamics [24,25,26,27]. Furthermore, the wind-tunnel tests of an aeroelastic model [19,20,21,22,23] and/or full-scale measurements [8,18] are expected for important bridges to provide added confidence on their performance under strong winds and to advance the theoretical analysis of the wind-induced vibrations of bridges. Compared to the wind-tunnel testing of the aeroelastic model, few data of full-scale measurement for cable-stayed bridges under construction subjected to strong winds, especially for the extreme double-cantilever and single-cantilever states, are available.

This study concentrates on the strong wind characteristics and associated buffeting response of a cable-stayed bridge during the extreme single-cantilever state. The wind velocities and accelerations at several locations along the bridge girder were measured by a wireless monitory system. The wind turbulence parameters of a strong typhoon and of monsoon, including turbulence intensities, gust factors, turbulence integral scales, and wind spectra are compared with recommendations in regional structural codes. Furthermore, the correlation between vertical and horizontal accelerations of bridge girder and strong wind velocities is analyzed. The buffeting responses of the bridge are then evaluated by applying the measured turbulence parameters, and the numerical results are compared with the field measurement accelerations of the bridge.

## 2. Full-Scale Measurement Set-Ups

### 2.1. Cable-Stayed Bridge and Its Surrounding Topography

**Figure 1:**
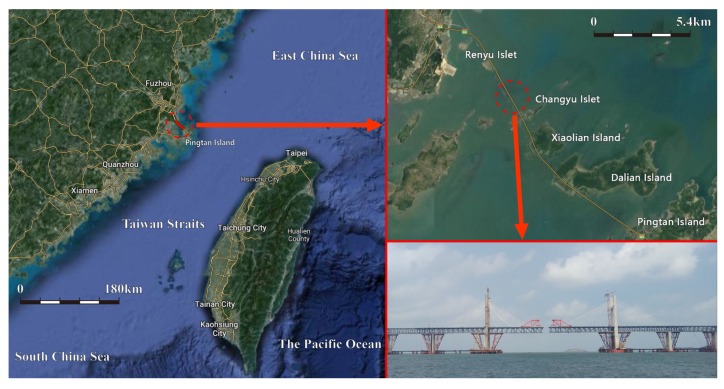
Bridge and its location.

A 364 m sea-crossing cable-stayed bridge under construction on the southeast coast of China is taken as the selected bridge. More specifically, the bridge is located at the northwest side of Taiwan Strait, and hence subjected to both strong typhoons and monsoons. This region, in fact, is among the extremely strong wind areas along the southeast coast of China (JTG/T 3360-01-2018 [28]). In addition, the wind speed-up over strait is evident due to the unique geographic location. The bridge under construction and its surrounding topography are shown in Figure 1.

### 2.2. Wireless Monitoring System

**Figure 2:**
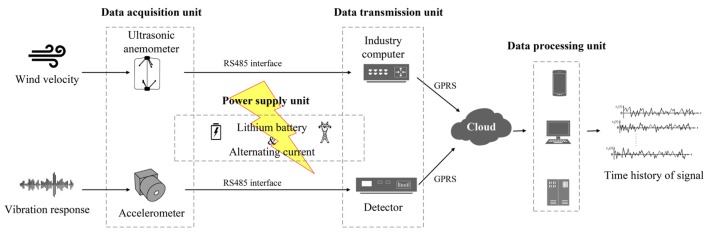
Architecture of the wireless monitoring system.

The unique strong wind climate of the Taiwan Strait site and the cable-stayed bridge under construction make it an interesting case study when investigating wind-induced dynamic responses of slender structures. For this reason, a WSHMS system has been designed and applied to capture the strong wind characteristics at the bridge site and buffeting response of cable-stayed bridge under construction. The monitoring system is mainly comprised of four units, namely data acquisition unit, data transmission unit, data processing unit and power supply unit. The architecture of the monitoring system is shown in Figure 2.

**Figure 3:**
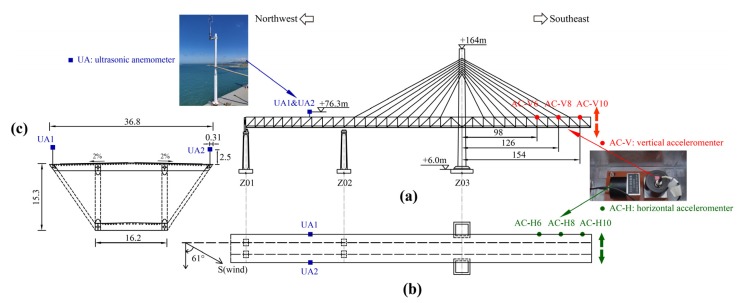
Arrangement of measurement sensors at the bridge under extreme single-cantilever state: (**a**) Elevation view; (**b**) Plan view; (**c**) Cross section of the bridge girder.

The data acquisition unit contains two WindMaster Pro triaxle ultrasonic anemometers (1561-PK-020/W, Gill Co. Ltd., Lymington, UK) collecting the wind velocities and six accelerometers (941 B, Institute of Engineering Mechanics, Harbin, China) on the girder surface capturing acceleration responses of the truss girder in both vertical and horizontal directions. The location of measurement sensors on the truss girder upper surface of the examined bridge is given in Figure 3. The two ultrasonic anemometers are installed at the top of 2.5 m tall iron column with an elevation of 76.3 m above the sea level. It should be noted that the interference effect from the truss girder is relatively smaller compared to that from the box girder [18]. Accordingly, the field measurements from the ultrasonic anemometers in this study can well present the oncoming wind characteristics. Three pairs of accelerometers are installed on the girder surface, at the distances of 98, 126 and 154 m from the center of the pylon, respectively. The wind direction is then defined south as 0° along an anticlockwise direction varying from 0° to 359.9°, and its resolution is 0.1°. The measuring range of the anemometer is from 0 to 65 m/s, and the measurement accuracy of the wind velocity is 0.001 m/s. The measurement range of accelerometers is ±20 m/s^2^ with the frequency measurement resolution of 5 × 10^−6^ m/s^2^, and the frequency band is 0.25–80 Hz. The sampling frequencies of wind gusts and acceleration of response were selected to be 4 Hz and 51.2 Hz, respectively.

The data transmission unit contains an industry-computer (F4931, Four-Faith Communication Technology Co. Ltd., Xiamen, China) and a 24-bit network distributed multi-function detector (NV3090A, Orient Institute of Noise & Vibration, Beijing, China). The wind and acceleration data are collected in real-time by the industry-computer and the detector, respectively. Both industry-computer and detector transmit the data to the data processing unit via the same router with GPRS simultaneously. The total data traffic per month is 2 GB, the data package of 500 Mb for each mobile phone card (China Telecom) has been ordered to satisfy the data traffic demand.

The intelligent cloud system (DASP-MTS, Orient Institute of Noise & Vibration) was designed and applied to store and treat the measured data. GPRS presents promising features of broad cover scope, high-speed and real-time transmission. These features can sufficiently satisfy the demand for transmitting measured data from the high-frequency anemometer through the GPRS network.

The rated power of each ultrasonic anemometer about 0.66 watts. The rated power of industry computer, detector and router are around 18, 25 and 3 watts, respectively. Therefore, the total power of the temporary monitoring system is less than 50 watts and was power supplied by on-site AC power. On the other hand, an uninterruptible power supply (ThinkPow, Jesen Electronic Co. Ltd., Guangzhou, China) was installed in case of the power outage during the typhoon, which can supply the power for 10 days independently.

## 3. Strong Wind Characteristics

### 3.1. Mean Wind Velocity and Direction

**Figure 4:**
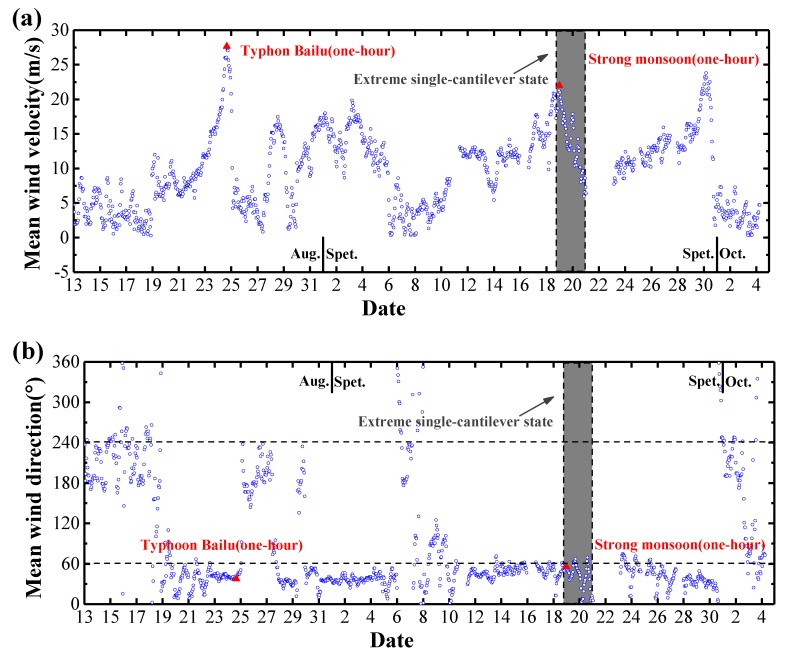
Measured mean wind velocities and directions with a one-hour interval from August 13th to October 5th: (**a**) Mean wind velocity; (**b**) Mean wind direction.

Figure 4 shows the measured mean wind velocities and directions with a one-hour interval from 13 August 2019 to 5 October 2019. Six strong wind events occurred during the period. Among them, Typhoon Bailu formed to the west of the Mariana Islands on August 20th and gradually intensified over the Philippine Sea. Typhoon Bailu made landfall over Taiwan on August 24th, and it entered the Taiwan Strait until making landfall over Dongshan County of Fujian at 07:25 (Beijing Time, hereafters) on August 25th. The investigated bridge was located approximately 320 km northeast of the landing center of Typhoon Bailu and the high wind velocities heavily affected the bridge construction and caused the shut-down for a week. In addition, the extreme single-cantilever state of the bridge lasted from 18:00 of September 18th to 24:00 of September 20th, and a very strong monsoon event occurred during this period (as shown in Figure 4). In this context, two hourly wind samples from Typhoon Bailu (16:00–17:00 of August 24th) and the strong monsoon (0:00 to 1:00 of September 19th), respectively, are selected to investigate the turbulence characteristics at the bridge site, and the associated buffeting response of bridge girder during the extreme single-cantilever state will be discussed in Section 4.

It was found that the selected two hourly-mean wind velocities are about 27.7 m/s and 22.0 m/s respectively. The hourly-mean azimuth angles of horizontal wind velocity are 37.4° and 55.5° respectively, and the azimuth is zero when the wind blows from the due north and 90° when the wind is from the due east. Furthermore, the mean wind yaw angle, defined as the horizontal angle between the direction of the mean wind and the normal to the bridge alignment, is zero when the azimuth angle is 61° or 241° (as shown in Figure 4). The average yaw angles of hourly mean wind measured are approximately 23.6° and 5.5°, respectively. It is noted that the wind azimuth angle at the bridge site is slightly close to 61°. Hence, the measured wind at UA2 that is located at the southeast side may be significantly disturbed by the bridge girder itself. Accordingly, only measured winds at UA1 are analyzed in this study. The wind records from full-scale measurements may present non-stationary features, and the discrete wavelet transform (DWT), empirical mode decomposition (EMD) and other numerical techniques are possible tools to identify the time-varying mean wind velocity. When time series of 10 min duration are used to analyze the wind turbulence parameters, removing the linear trend is the “standard process”, and it was also adopted with a 10-min moving average in the present study to obtain the overall trends of turbulence intensity, gust factor and turbulence integral scale, as well as wind velocity power spectrum of strong winds [29,30].

### 3.2. Turbulence Intensity and Gust Factor

The wind information measured from the triaxle ultrasonic anemometer can be converted to the instantaneous fluctuating components in the longitudinal, lateral and vertical directions, respectively. The turbulence intensity is defined as the standard deviation of the fluctuating component divided by the mean wind velocity [31]:

(1) $$I_{a}=\frac{\sigma_{a}}{U},$$

where *I_a_* (*a* = *u*, *v*, *w*) is the turbulence intensity in the longitudinal, lateral and vertical directions, respectively; *σ_a_* is the corresponding standard deviation; *U* is the mean wind velocity over a time interval of 10 min.

The gust factor represents the ratio of the average maximum wind velocity in the gust duration to the mean wind velocity [31]:

(2) $$G_{a}=\{\begin{array}{c} 1+\max(\mathrm{abs}(a(t_{g})))/U,\;a=u\\ \max(\mathrm{abs}(a(t_{g})))/U,\;a=v,w \end{array},$$

where *G_a_* is the gust factor in the longitudinal, lateral and vertical directions, respectively; *t_g_* is the gust duration and is set to 3 s.

**Figure 5:**
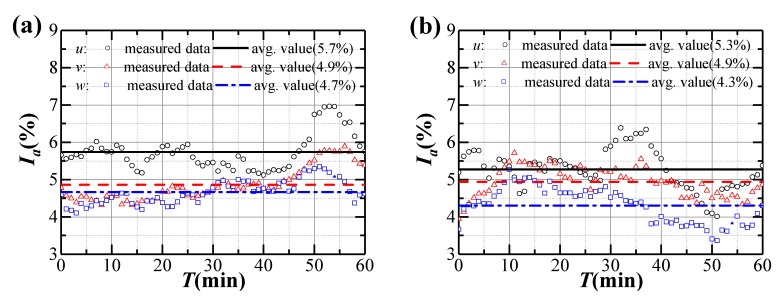
Variation of turbulence intensities during strong winds: (**a**) Typhoon Bailu; (**b**) Strong monsoon event.

**Figure 6:**
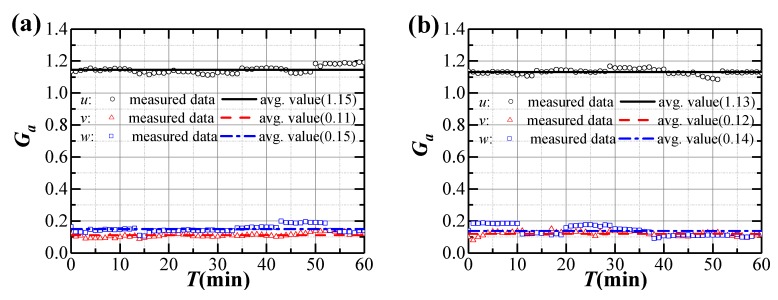
Variation of gust factors during strong winds: (**a**) Typhoon Bailu; (**b**) Strong monsoon event.

Variation of turbulence intensities and gust factors during Typhoon Bailu and strong monsoon event are shown in Figure 5 and Figure 6. Both the turbulence intensity and gust factor in the longitudinal, lateral and vertical directions present fluctuations. The measured longitudinal average turbulence intensity and gust factor during Typhoon Bailu are 5.7% and 1.15, respectively, which are close to those measured during the strong monsoon (5.3% and 1.13, respectively). The turbulence intensities and gust factors in the lateral and vertical directions during Typhoon Bailu and strong monsoon event are also similar. Hence, it seems that the turbulence intensities and gust factors during Typhoon and monsoon events share a considerable amount of similarity. This observation is consistent with the results presented in Masters et al. [32]. One possible reason is that the bridge site is relatively far from the landfall center of Typhoon Bailu (approximately 320 km).

The statistics of measured turbulence intensities and gust factors are shown in Table 1. It can be seen that the measured average value of longitudinal turbulence intensity during Typhoon Bailu and strong monsoon event is 5.5%, smaller than those specified in JTG/T 3360-01-2018 [28] for terrain category A (11.0%) or in EN 1991-1-4:2005 (9.8%) [33] and AIJ 2004 (11.9%) [34]. However, the longitudinal turbulence intensity is relatively close to the value interpolated from the measured data (6.5%) at Mount Wangye (near to the bridge site) wind measurement tower [4]. The measured average ones in the lateral and vertical directions behave similarly. The turbulence intensity ratio between lateral and longitudinal direction is 0.89, very close to the recommended value of 0.88 in JTG/T 3360-01-2018 [28], but the ratio between vertical and longitudinal direction is higher than the recommended value in Chinese code. The average longitudinal gust factor is 1.14, and is also smaller to the recommended values in various regional codes.

### 3.3. Turbulence Integral Scale

**Figure 7:**
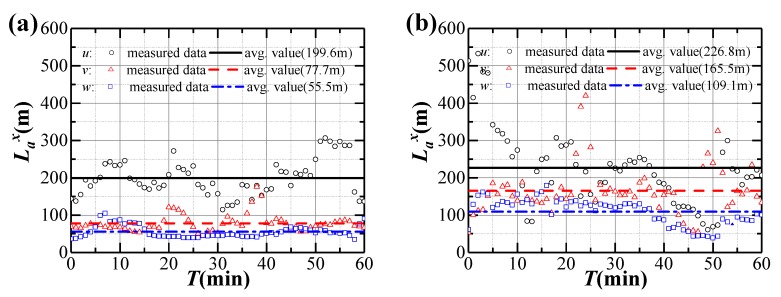
Variation of integral length scales during strong winds: (**a**) Typhoon Bailu; (**b**) Strong monsoon event.

Turbulence integral scales are measurements of the average size of the turbulent eddies of the flow, and it can be calculated using the autocorrelation method as follows [31]:

(3) $$L_{a}^{x}=U\cdot T_{a}=\frac{U}{\sigma_{a}^{2}}\cdot \int_{0}^{{}_{\infty }}R(\tau )d\tau$$

where *L_a_^x^* is the turbulence integral scale of fluctuating component *a* in the longitudinal direction, *T_a_* is the corresponding time scale according to Taylor’s hypothesis of convected “frozen turbulence”, and *R(τ)* is the autocorrelation function of the fluctuating component *a*. It should be noted that the up-limit of *τ* will be cut by *R(τ)* less than 0.05 [35]. The measured integral length scales during Typhoon Bailu and strong monsoon event are shown in Figure 7. The collected data present fluctuations, particularly for the longitudinal direction. On the other hand, the average longitudinal length scales during Typhoon Bailu and strong monsoon event are 199.6 and 226.8 m, respectively. They are close to the value of 211.4 m that is recommended in European code (EN 1991-1-4: 2015) [33], and are larger than 140 m in JTG/T 3360-01-2018 [28] and 162.8 m in AIJ 2004 [34]. The average lateral and vertical length scales during strong monsoon event are 165.5 and 109.1 m, and they are larger than those during Typhoon Bailu.

### 3.4. Wind Velocity Power Spectrum

The power spectral density (PSD) represents the distribution of the kinetic energy of the wind eddies in the frequency domain and is often presented in a normalized form. For longitudinal velocity component *u*, the von Karman spectrum [36], Kaimal spectrum [37], Simiu spectrum [28,31] and Harris (Modified Davenport) spectrum [38] are respectively expressed as follows:

(4) $$\frac{fS_{u}}{u_{*}^{2}}=\frac{4\beta_{u}^{2}\;(f_{z}L_{u}^{x}/z)}{{\left(\;1+70.78\;(f_{z}L_{u}^{x}/z{)}^{2}\right)}^{\;5/6}},$$

(5) $$\frac{fS_{u}}{u_{*}^{2}}=\frac{105\;f_{z}}{{\left(\;1+33\;f_{z}\right)}^{\;5/3}},$$

(6) $$\frac{fS_{u}}{u_{*}^{2}}=\frac{200\;f_{z}}{{\left(\;1+50\;f_{z}\right)}^{\;5/3}},$$

(7) $$\frac{fS_{u}}{u_{*}^{2}}=\frac{4\;[f_{z}(1800/z){\left(z/10\right)}^{\alpha }]}{{\left\{2+\;[f_{z}(1800/z){\left(z/10\right)}^{\alpha }{]}^{2}\right\}}^{\;5/6}},$$

where *f_z_*= *fz*/*U* is the non-dimensional frequency; *f* is the engineering frequency (Hz); *z* is the height of UA1 and is 76.3 m; *μ_⁎_* = *σ_a_*/*β_a_* is the friction velocity; *β_u_* is taken to 2.542 at the sea or coastal area (*z*_0_ = 0.01) by Bietry et al. [39], and *β_v_* and *β_w_* are 0.89 *β_u_* and 0.81 *β_u_* respectively as discussed in Section 3.2; α is the exponent coefficient of the power law of wind profile and is suggested to 0.12 at the sea or coastal area according to the JTG/T 3360-01-2018 [28].

For lateral velocity component *v*, the von Karman spectrum [36], Kaimal spectrum [37] and Simiu spectrum [31] are respectively expressed as follows:

(8) $$\frac{fS_{v}}{u_{*}^{2}}=\frac{4\beta_{v}^{2}\;(f_{z}L_{v}^{x}/z)[1+755.2{\left(f_{z}L_{v}^{x}/z\right)}^{2}]}{{\left[1+283.2\;(f_{z}L_{v}^{x}/z{)}^{2}\right]}^{\;11/6}},$$

(9) $$\frac{fS_{v}}{u_{*}^{2}}=\frac{17\;f_{z}}{{\left(\;1+9.5\;f_{z}\right)}^{\;5/3}},$$

(10) $$\frac{fS_{v}}{u_{*}^{2}}=\frac{15\;f_{z}}{{\left(\;1+9.5\;f_{z}\right)}^{\;5/3}},$$

For vertical velocity component *w*, the von Karman spectrum [36], Kaimal spectrum [37], Panofsky spectrum [28,40] and Irwin spectrum [41] are similar to the above formula with different coefficients:

(11) $$\frac{fS_{w}}{u_{*}^{2}}=\frac{4\beta_{w}^{2}\;(f_{z}L_{w}^{x}/z)[1+755.2{\left(f_{z}L_{w}^{x}/z\right)}^{2}]}{{\left[1+283.2\;(f_{z}L_{w}^{x}/z{)}^{2}\right]}^{\;11/6}},$$

(12) $$\frac{fS_{w}}{u_{*}^{2}}=\frac{2\;f_{z}}{1+5.3\;f_{z}^{5/3}},$$

(13) $$\frac{fS_{w}}{u_{*}^{2}}=\frac{6\;f_{z}}{{\left(\;1+4\;f_{z}\right)}^{\;2}},$$

(14) $$\frac{fS_{w}}{u_{*}^{2}}=\frac{2\beta_{w}^{2}\;(0.8f_{z})[1+188.8{\left(0.8f_{z}\right)}^{2}]}{{\left[1+70.78\;(0.8f_{z}{)}^{2}\right]}^{\;11/6}},$$

**Figure 8:**
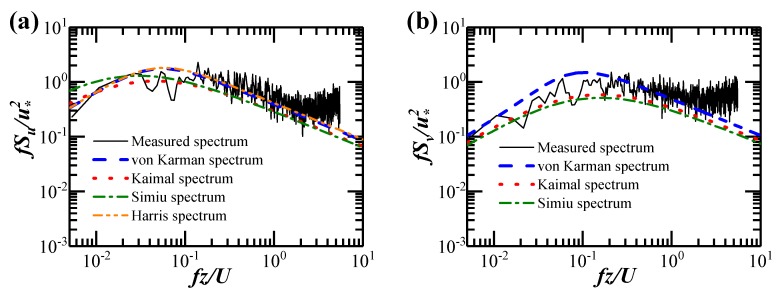

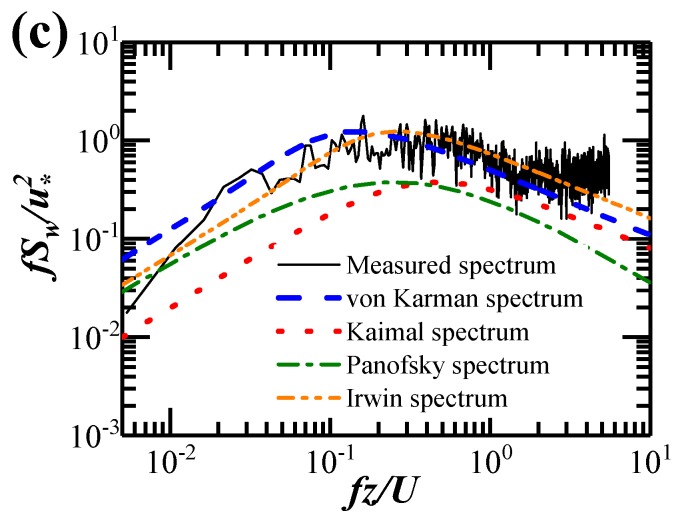
Wind spectra obtained via the field measurement: (**a**) Longitudinal velocity; (**b**) Lateral velocity; (**c**) Vertical velocity.

Figure 8 displays the longitudinal, lateral, and vertical wind spectra during Typhoon Bailu. The Welch method was adopted in the spectral analysis of the one-hour strong wind with 14,400 samples, which was divided into 15 sub-segments with an overlapped length of 3.75 minutes between two neighboring sub-segments. The block size for the FFT was equal to 2048, and the Hamming window was used in the spectral analysis of each sub-segment to decrease the leakage of signals in the frequency domain from one band to another. The results depicted in Figure 8 suggest that the wind spectra obtained via the field measurements can be approximated by the von Karman spectra. This phenomenon is in accordance with Li et al. [42]’s observation during the passage of Typhoon Sally in 1996. The longitudinal and vertical ones also fit well with the Harris and Irwin spectra respectively.

## 4. Buffeting Response of the Bridge during the Extreme Single-Cantilever State

### 4.1. Acceleration Responses

**Figure 9:**
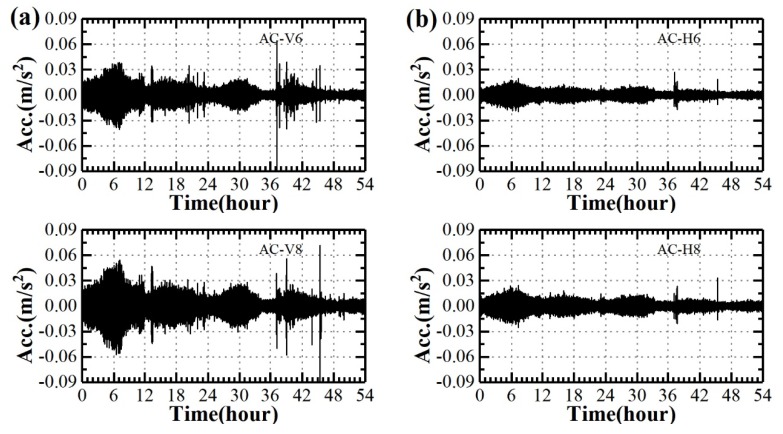

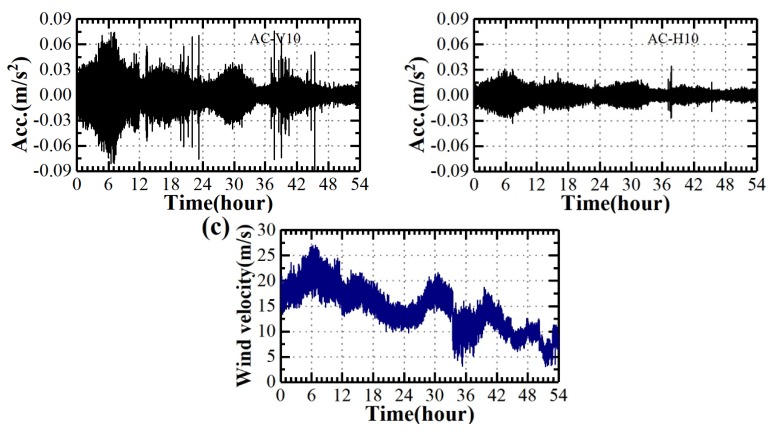
Time histories of accelerations of bridge girder and corresponding wind velocity: (**a**) Vertical acceleration; (**b**) Horizontal acceleration; (**c**) Wind velocity.

The extreme single-cantilever state lasts from 18:00 of September 18th to 24:00 of September 20th (54 h), and this period is selected to analyze the bridge buffeting response. Figure 9 illustrates the time histories of vertical and horizontal acceleration responses of the bridge girder, and the corresponding wind velocities are also depicted in this figure. Since the interested frequency of the bridge structure is lower than 2 Hz (for example, the tenth natural frequency is 1.744 Hz), the accelerometer signals were low-passed filtered with a cut-off frequency of 4 Hz. The vertical acceleration is much larger than the horizontal acceleration, and the vibration patterns are also different from each other. The vertical vibration responses of AC-V10 are larger than those of AC-V6 and AC-V8, and the vertical vibration responses of AC-V6 are the smallest due to the vibration mode shape. The relationships among horizontal vibration responses of AC-6H, AC-8H and AC-10H behave similarly. It is also observed that there is an evident correlation between instantaneous values of wind velocity and bridge girder acceleration. To reduce the interference of possible construction operation on the buffeting responses of the bridge, only acceleration responses against strong winds (i.e., the 10-min wind velocity is larger than 12 m/s) are considered in the following buffeting response analysis.

**Figure 10:**
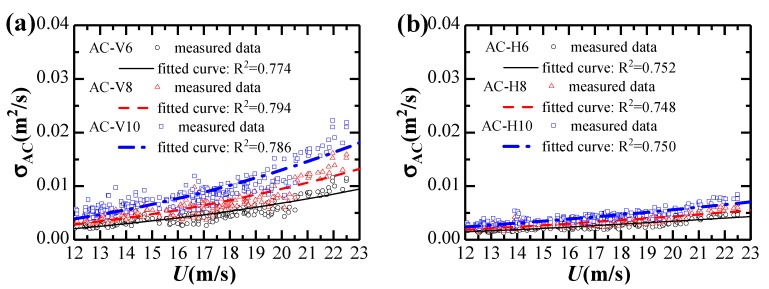
Dependence of acceleration responses on strong winds: (**a**) Vertical; (**b**) Horizontal.

Considering the yaw angle variation during the extreme single-cantilever state, the effects of skew winds are not considered in this study. Variations of standard deviations of vertical and horizontal accelerations against strong winds are given in Figure 10.

The standard deviations of accelerations in vertical and horizontal directions generally increase with the 10-min mean wind velocity and could be fitted by the empirical relationship as:

(15) $$\sigma_{\mathrm{AC}}=10^{p}\cdot U^{q},$$

where *p*, *q* are the fitting parameters determined from the measured data.

Fitting parameters for the relationship between acceleration responses and the 10-min mean wind velocity are listed in Table 2. It can be observed that Equation (15) fits the results in Figure 10a,b well. The vibration response in the horizontal direction is significantly smaller than in the vertical direction. The vertical acceleration responses increase approximately in proportion to the 2.36th power of mean wind velocity, while the horizontal acceleration response increases approximately proportionally to the 1.66th power of mean wind velocity.

### 4.2. Spectra and Natural Frequencies

**Figure 11:**
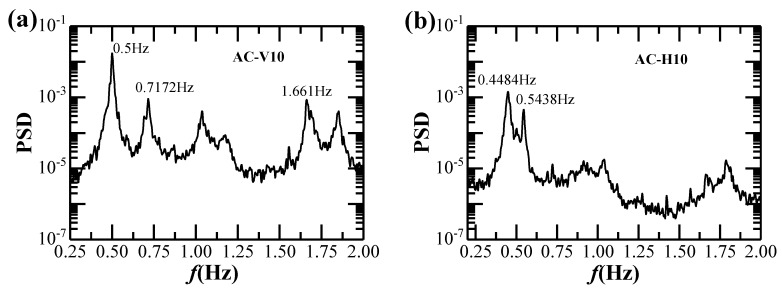
Acceleration spectra of the bridge girder: (**a**) Vertical; (**b**) Horizontal.

The auto spectra of the vertical and horizontal acceleration responses of the bridge are obtained from the response time histories. Figure 11 shows only the spectra of vertical and horizontal acceleration responses measured from the AC-V10 and AC-H10 during the strong monsoon as an example (as discussed in Section 3.1). The first three vertical modal frequencies form the finite element model (FEM) are 0.5097 Hz, 0.8201 Hz and 1.6338 Hz, while the corresponding values identified from the vertical acceleration spectra are 0.5 Hz, 0.7172 Hz and 1.661 Hz. Furthermore, the first and second horizontal modal frequencies from FEM are 0.4715 Hz and 0.6592 Hz, while the corresponding identified values from the horizontal acceleration spectra are 0.4484 Hz and 0.5438 Hz, respectively. It is noted that the modal frequencies from FEM and from full-scale measurements are very close. The relative difference between the first vertical frequencies is around 5% whilst that of the first horizontal frequencies is less than 2%.

### 4.3. Comparison of Buffeting Analysis between Field-Measurements and Numerical Analysis

A complete quadratic combination (CQC) approach [43] is performed to calculate the vertical and horizontal bridge girder buffeting accelerations in the case of normal wind. The drag coefficient of the bridge girder measured from the wind tunnel tests at Central South University is 0.6452 at the zero wind angle of attack with respect to the girder height of 15.3 m, and the lift and moment coefficients are 0.0308 and 0.1065, respectively with respect to the girder width of 36.8 m. The first derivatives of the drag, lift, and moment coefficients (*C_D_′*, *C_L_′*, *C_M_′*) with respect to zero wind angle of attack are 0.3266, 4.3144, and 0.6088, respectively. In the simulation of the self-excited forces, the flutter derivatives for horizontal direction are computed based on the quasi-steady theory and the results from a similar bridge girder (Øresund Strait Bridge) is utilized for other directions [44]. The first 10 natural modes are taken into consideration in the buffeting response analysis, and a frequency interval about 0.001 is used within the range from 0.001 to 2 Hz. The structural damping ratios of the natural modes are assumed to be 0.005 for all modes under consideration [28]. The decay coefficients utilizing Davenport’s model of the vertical and spanwise coherence of longitudinal and vertical wind fluctuations are assumed to be 7 [28]. Both the unity function and the Sears function [45] for all frequency range are considered as the aerodynamic admittance of the bridge girder.

**Figure 12:**
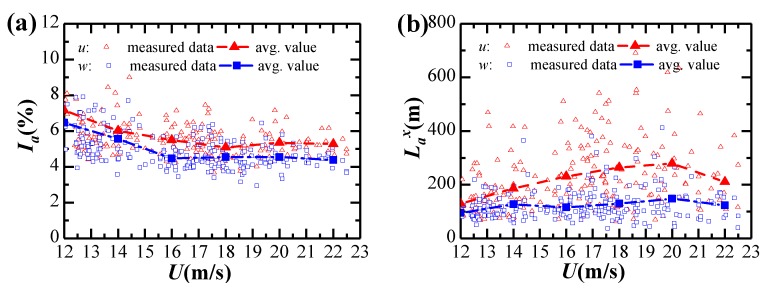
Dependence of turbulence intensity and turbulence integral scale on mean wind velocity: (**a**) Turbulence intensity; (**b**) Turbulence integral scale.

The buffeting analysis was performed in three analysis conditions, as shown in Table 3. The measured longitudinal and vertical turbulence intensities and turbulence integral scales with the mean wind velocities are shown in Figure 12. The turbulence intensity approximately decreases with the mean wind velocity, while the turbulence integral scale approximately increases with the mean wind velocity. Both turbulence parameters proposed by design code [28] and measured ones in Figure 12 are respectively considered in Case 1 and Case 2 analysis conditions using the unity aerodynamic admittance. Case 3 considers the Sears aerodynamic admittance under the same condition of turbulence parameters as Case 2.

**Figure 13:**
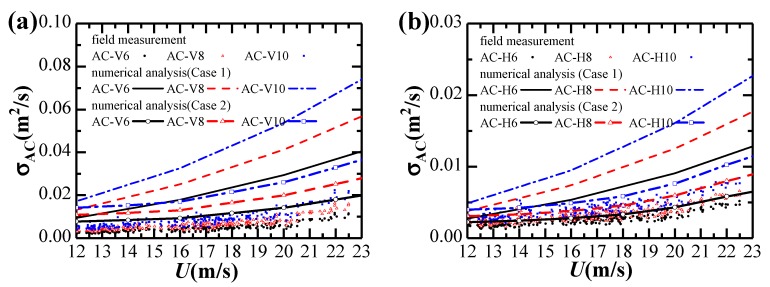
Comparison of standard deviations of acceleration response obtained by field measurements and numerical analysis in Case 1 and Case 2: (**a**) Vertical; (**b**) Horizontal.

Figure 13 shows the standard deviations of acceleration response obtained by field measurements and numerical analysis in Case 1 and Case 2 analysis conditions. Both buffeting responses from the field measurement are generally smaller than numerical analysis results. However, the calculated results using turbulence parameters from the design code give an overestimation for the buffeting response, while the calculated results using measured turbulence parameters provide a good estimate of buffeting response, especially for the horizontal response. Therefore, it is of significance to analyze the strong wind characteristics at the bridge site for a better prediction of the wind-induced dynamic response of the bridge.

**Figure 14:**
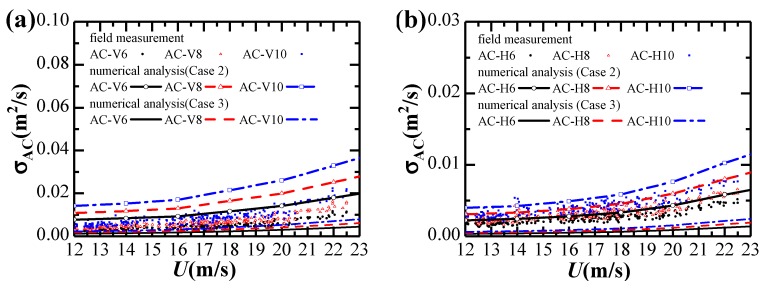
Comparison of standard deviations of acceleration response obtained by field measurements and numerical analysis in Case 2 and Case 3: (**a**) Vertical; (**b**) Horizontal.

Figure 14 shows the standard deviations of acceleration response obtained by field measurements and numerical analysis in Case 2 and Case 3 analysis conditions. The calculated results based on the Sears aerodynamic admittance are smaller than the experimental buffeting responses, which indicates that the buffeting response analysis using the Sears aerodynamic admittance may significantly underestimate the buffeting response. It also can be observed that bridge girder aerodynamic admittance has an essential influence on the buffeting response analysis. The discrepancies between the field-measurements and numerical results in Case 2 and Case 3, especially in the vertical direction, are attributed to a number of factors, among which aerodynamic admittances might be essential, and more attention should be given to accurately and reliably identify its values in the wind tunnel experiment and CFD in the future.

## 5. Conclusions

This study carried out a detailed full-scale investigation on the strong wind characteristics at a cable-stayed bridge site and associated buffeting response of the structure during construction, using a filed monitoring system. The following conclusions were obtained:

1. Wind characteristics during typhoon and monsoon events share a considerable amount of similarity, and can be described as the input turbulence parameters for the current wind-induced vibration theory.
2. The longitudinal turbulence integral scales are consistent with those in regional structural codes, while the turbulence intensities and gust factors are less than the recommended values. The wind spectra obtained via the field measurements can be well approximated by the von Karman spectra.
3. The vertical acceleration responses of the bridge girder at the extreme single-cantilever state are larger than those in the horizontal direction, and the increasing tendencies with mean wind speeds are also different from each other.
4. The buffeting analysis results using wind turbulence parameters proposed in the design code and the unity aerodynamic admittance of the bridge girder are on the conservative side, and they can be used in the preliminary phase of the design of cable-stayed bridges.
5. The buffeting analysis results using measured wind turbulence parameters provide a good estimate of buffeting response, especially for the horizontal response. Accordingly, it is important to measure wind velocities at the bridge site for reasonable design and construction of cable-stayed bridges.
6. The discrepancies between the field-measurements and numerical results may mainly be attributed to the bridge girder aerodynamic admittance, and hence more attention should be given to accurately and reliably identify its values using wind tunnel experiments or computational fluid dynamics simulations in the future.

## Figures and Tables

**Table 1:** Statistics of measured average turbulence intensities and gust factors.

Wind Field	*I_u_*(%)	*I_v_*(%)	*I_w_*(%)	*I_u_*:*I_v_*:*I_w_*	*G_u_*	*G_v_*	*G_w_*
Typhoon Bailu	5.7	4.9	4.7	1:0.85:0.81	1.15	0.11	0.15
Strong monsoon	5.3	4.9	4.3	1:0.94:0.82	1.13	0.12	0.14
Average values	5.5	4.9	4.5	1:0.89:0.81	1.14	0.12	0.14
JTG/T 3360-01-2018 [28]	11.0	9.6	5.5	1:0.88:0.5	1.24		
EN 1991-1-4:2005 [33]	9.8				1.30		
AIJ 2004 [34]	11.9				1.35		

**Table 2:** Fitting parameters for the relationship between acceleration responses and wind velocities.

Fitting Parameters	AC-V6	AC-V8	AC-V10	AC-H6	AC-H8	AC-H10
*p*	−5.19	−5.12	−4.97	−4.63	−4.53	−4.43
*q*	2.32	2.38	2.37	1.66	1.66	1.67

**Table 3:** Numerical analysis cases.

Case	Conditions	
	Wind Turbulence Parameters	Bridge Girder Aerodynamic Admittance
Case 1	Wind spectrum, turbulence intensity and turbulence integral scale proposed by design code	Unity function
Case 2	von Karman spectrum, measured turbulence intensity and turbulence integral scale with mean wind velocity	Unity function
Case 3	von Karman spectrum, measured turbulence intensity and turbulence integral scale with mean wind velocity	Sears function
